# *Clostridium perfringens* in central Colombia: frequency, toxin genes, and risk factors

**DOI:** 10.1186/s13099-024-00629-5

**Published:** 2024-07-04

**Authors:** Anny Camargo, Laura Bohorquez, Diana Paola López, Atilio Ferrebuz-Cardozo, José Castellanos-Rozo, Javier Díaz-Ovalle, Mariana Rada, Milena Camargo, Juan David Ramírez, Marina Muñoz

**Affiliations:** 1 Centro de Investigaciones en Microbiología y Biotecnología-UR (CIMBIUR), Facultad de Ciencias Naturales, Universidad del Rosario, Bogotá, Colombia; 2 Universidad de Boyacá, Tunja, Colombia; 3 Centro de Tecnología en Salud (CETESA), Innovaseq SAS, Funza, Cundinamarca Colombia; 4 Molecular Microbiology Laboratory, Department of Pathology, Molecular and Cell-Based Medicine, Icahn School of Medicine at Mount Sinai, New York, NY 10029 USA; 5 Instituto de Biotecnología-UN (IBUN), Universidad Nacional de Colombia, Bogotá, Colombia

**Keywords:** *Clostridium perfringens*, Symptomatic, Asymptomatic, Toxins, Risk factors

## Abstract

*Clostridium perfringens* is an opportunistic bacterium that causes intestinal diseases in both humans and animals*.* This study aimed to assess the frequency of *C. perfringens* and the presence of toxin-encoding genes in fecal samples from individuals with or without gastrointestinal symptoms in the Department of Boyacá, Colombia. Additionally, risk factors associated with carriage and disease development were analyzed. A total of 114 stool samples were analyzed using a molecular test based on specific polymerase chain reaction (PCR) targeting 16S-rRNA and alpha toxin (*cpa*) genes. For individuals with a positive result for the PCR test, stool samples were cultured on Tryptose Sulfite Cycloserine (TSC) agar. Two to five colonies forming units were selected based on phenotypic characteristics, resulting in 56 bacterial isolates. These isolates were then analyzed for toxin-coding genes associated with gastrointestinal diseases. In addition, sociodemographic and clinical data from 77 individuals were also analyzed. The overall frequency of *C. perfringens* was 19.3% (n = 22/114). The detection frequency in 77 individuals with clinical data was 16.6% (n = 5/30) among symptomatic individuals and 21.2% (n = 10/47) among asymptomatic individuals. All 56 isolates obtained carried the *cpa* gene, while *cpb2* was present in 10.7% (n = 6/56); *cpe* and *cpb* genes were not detected. Notably, diabetes and autoimmune diseases are significantly associated with an increased risk of *C. perfringens* detection (adjusted OR 8.41: 95% CI 1.32–35.89). This study highlights an elevated frequency of *C. perfringens* and the presence of the *cpb2* gene in asymptomatic individuals compared with their symptomatic counterparts. These findings offer insights into the distribution and virulence factors of *C. perfringens* at a micro-geographical level. This information supports the need for developing tailored prevention strategies based on local characteristics to promote active surveillance programs based on molecular epidemiology.

**Supplementary Information:**

The online version contains supplementary material available at 10.1186/s13099-024-00629-5.

## Introduction

*Clostridium perfringens*, an opportunistic enteropathogenic bacterium affecting humans and animals, has been linked to multiple intestinal and systemic diseases, including food poisoning, antibiotic-associated diarrhea (AAD), and intestinal necrosis [1, 2]. Its ability to produce toxins contributes to the development of severe infections [3]. In many instances, the factors influencing or mitigating this risk in individual patients still need to be clarified. Some studies have indicated that *C. perfringens* carriage, along with host-associated risk factors such as the use of antacids [1] and older age (> 50 years) [4], may predispose individuals to infection development.

Understanding predisposing host factors in specific contexts is essential for designing tailored preventive and therapeutic strategies, thereby enhancing public health efforts to address the incidence and impact of these diseases more effectively. Similarly, risk factors associated with the microorganism, such as a rapid growth rate, production of high-temperature resistant spores, the release of toxins, and antibiotic resistance mechanisms, influence the development of severe infections that are difficult to control [5].

The Cpa, Cpe, Cpb, and Cpb2 toxins are linked to gastrointestinal disease [6], highlighting the importance of their routine surveillance and the implementation of improved control measures for circulating strains carrying these toxins. Toxin of *C. perfringens* also includes perfringolysin O (PfoA), enterotoxin (Cpe), and necrotic enteritis B-like toxin (NetB) [3]. These toxins form a heterogenous combination of toxin-encoding genes classified into seven toxinotypes (A to G), each associated with a specific disease in a particular host [5, 6]. Therefore, molecular epidemiology studies could contribute to understanding the clinical, sociodemographic, and biological factors that influence the development of diseases caused by *C. perfringens* and other pathogens.

Although molecular epidemiology has explored the detection of *C. perfringens* in symptomatic individuals in Europe, Asia, and North America, reporting detection frequencies ranging from approximately 5 to 20% in cases of antibiotic-associated diarrhea (AAD) and sporadic non-foodborne diarrhea [1, 7–11], comparative studies between the frequency of detection in asymptomatic and symptomatic individuals, as well as information about predisposing factors, especially in developing countries, are limited.

In Colombia, a 32.7% infection rate was observed in patients with diarrhea, but this data lacks detailed sociodemographic and clinical information, hindering the establishment of significant clinical associations [12]. The absence of relevant data poses challenges to effectively contributing to implementing public health measures aimed at reducing the disease burden.

Therefore, we aimed to identify the presence of *C. perfringens* in fecal samples from individuals with gastrointestinal symptoms and asymptomatic individuals in a central region of Colombia. Additionally, we identified potential circulating toxins and collected relevant sociodemographic and clinical data to understand the characteristics of the population carrying *C. perfringens*. By exploring these dimensions, our goal was to contribute to understanding the relationship between the presence of *C. perfringens*, toxin circulation, and population characteristics. The results of this study contribute to the local understanding of the molecular epidemiology of *C. perfringens* in Colombia.

## Methods

### Study population

Between May and September 2022, 114 fecal samples were collected from adults aged between 26 and 84 years. These samples were obtained from individuals seeking healthcare services at three hospitals in the Department of Boyacá, Colombia (Figure supplementary 1). Participant selection included individuals with gastrointestinal symptoms such as diarrhea, abdominal pain, fever, or vomiting, who were referred for stool sample collection due to their symptoms. Asymptomatic participants were also included in the study, selected as part of routine examinations, primarily screening tests (fecal occult blood test) due to risk factors such as advanced age (> 60 years), chronic diseases like diabetes, hypertension, hypothyroidism, gastritis, or autoimmune diseases (Supplementary Table 1).

Comprehensive data were collected from 77 out of the 114 participants, with a few choosing not to disclose information due to privacy concerns or time constraints in responding to the survey. This included sociodemographic details (gender, education level, access to clean water, food refrigeration, presence of animals at home, and age), as well as clinical information such as antibiotic consumption in the last three months and the presence of chronic diseases as a possible risk factor for the development of *C. perfringens* infectious diseases.

### DNA extraction and molecular detection of *C. perfringens*

DNA extraction from the 114 samples was performed from a 300 mg fecal sample aliquot using the Norgen fecal DNA isolation kit following the manufacturer's instructions. DNA concentration was measured with the NanoDrop/2000/2000c spectrophotometer (Thermo Fisher Scientific, Massachusetts, USA). Subsequently, *C. perfringens* was detected through conventional PCR using two primer sets targeting the genes: (i) *16S rRNA* gene and (ii) *cpa* gene. Both PCR reactions were carried out under conditions described in previous studies [13]. *C. perfringens* ATCC^®^ 13124 strain was included as a positive control. The presence of an amplified product for both genes, *16S rRNA* (product size 481 bp) and *cpa* (product size 400 bp) was considered a positive result for *C. perfringens.*

### Bacterial culture and identification of potential toxins

From samples that tested positive for *C. perfringens* during PCR, a second 100 uL aliquot of the sample was used for in vitro cultivation on Tryptose Sulfite Cycloserine agar (TSC), incubated for 24 h at 37 °C under anaerobic conditions following the methodology described by other authors [14]. Subsequently, 2 to 5 black colonies per sample were selected based on their morphological differences and lecithinase activity to capture the greatest possible diversity of isolates within the same individual. They were then checked by Gram staining. The biomass was increased on blood agar, and bacterial genomic DNA was extracted using Promega's Wizard genomic DNA purification kit. DNA concentration was measured with the NanoDrop/2000/2000c spectrophotometer (Thermo Fisher Scientific, Massachusetts, USA).

The presence of genes encoding Cpa, Cpe, Cpb, and Cpb2 toxins on *C. perfringens* isolates was evaluated by PCR using specific primer pairs detailed in Table 1. Amplification reactions were carried out in a 12.5 μL volume, with a template DNA volume of 1.5 μL (100 ng/μL), following the previously described conditions [13, 15]. PCR products were visualized through electrophoresis on a 1.5% agarose gel with a 100 bp molecular weight marker.

**Table 1:** Primers used in the PCR to detect toxins from *C. perfringens*

Toxin	Primer name	Sequence (5′–3′)	Gene	Product size (bp)	Ref
Cpa	CpaF CpaR	TGCATGAGCTTCAATTAGGT TTAGTTTTGCAACCTGCTGT	*Alpha-toxin*	400	[13]
Cpe	CpeL CpeR	GGGGAACCCTCAGTAGTTTCA ACCAGCTGGATTTGAGTTTAATG	*Entero-toxin*	506	[15]
Cpb	CpbL CpbR	TCCTTTCTTGAGGGAGGATAAA TGAACCTCCTATTTTGTATCCCA	*Beta-toxin*	611	[15]
Cpb2	Cpb2L Cpb2R	CAAGCAATTGGGGGAGTTTA GCAGAATCAGGATTTTGACCA	*Beta2—toxin*	200	[15]

### Statistical analysis

Given the distribution of the quantitative variable of age, we chose to use the median and reported its measure of dispersion, the interquartile range (IQR). The qualitative variables (detailed in Supplementary Table 1.), such as sex, presence of animals in the home, access to drinking water, hypertension, gastritis, diabetes, and hypothyroidism, were presented in terms of frequencies and percentages.

Conditional logistic regression models were employed to evaluate associations between C. perfringens detection, identified toxin, and sociodemographic and clinical variables. The model provided estimates of odds ratios (OR) in crude and adjusted form, accompanied by their 95% confidence intervals (CI). The first model considered was the detection of *C. perfringens*, while the second focused on detecting toxins genes (*cpa*, *cpe*, *cpb*, and *cpb2*) as the dependent variables. Independent variables for both models included age, presence of animals in the home, antibiotic use, access to drinking water, and comorbidities such as hypertension, gastritis, diabetes and autoimmune diseases, hypothyroidism, hypercholesterolemia, and any reported symptoms. Multicollinearity among covariates was assessed using variance inflation factor (VIF) and tolerance [16, 17]. All two-tailed statistical tests were performed using the STATA 14 program, with p values < 0.05 considered statistically significant.

## Results

This study involved collecting and analyzing 114 stool samples from individuals seeking care at three hospital centers in the Department of Boyacá, Colombia. A detailed survey obtained sociodemographic and clinical data from 77 participants (see Supplementary Table 1).

**Fig. 1:**
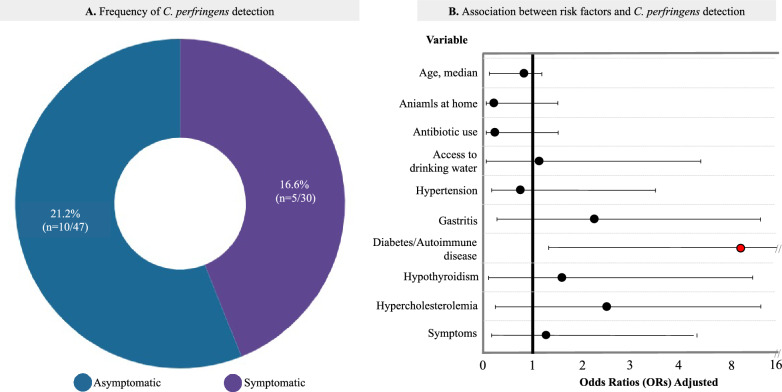
**A** Frequency of detection of *C. perfringens* in symptomatic (infected) vs asymptomatic (colonized) individuals. **B** Schematic representation of the adjusted Odds ratio (OR) together with its corresponding 95% confidence interval measures the strength of association between the risk factors evaluated and the detection of *C. perfringens*

Globally, the detection frequency of *C. perfringens* was 19.3% (n = 22/114). Most participants were women, comprising 64.9% (n = 50/77), with a mean age of 57.2 years, and 20.7% had completed secondary education. The frequency of detection in the 77 individuals with clinical data was 16.6% (n = 5/30) in symptomatic individuals, while in asymptomatic individuals, it was 21.2% (n = 10/47) (Fig. 1A**)**.

Among the symptomatic individuals, the average age was 56.6 years, 80.0% were women, and all were over 50. Additionally, 40.0% had consumed antibiotics in the last three months, lived with animals, and had hypertension as the most common comorbidity.

Asymptomatic individuals had an average age of 62.1 years, with 40% being women. Additionally, 20% had taken antibiotics in the last three months, and 40% lived with animals. Associated comorbidities included hypertension in 50% of symptomatic humans, diabetes and autoimmune diseases in 30%, hypercholesterolemia in 20%, and gastritis or hypothyroidism in 10%.

The analysis of associated comorbidities in the population revealed an increased odds ratio for diabetes and autoimmune diseases (adjusted OR 8.41: 95% CI 1.32–35.89) (Fig. 1B, Supplementary Table 2). No statistically significant associations were found between the variables and the group of patients infected versus colonized.

A total of 56 isolates of *C. perfringens* were obtained from 22 stool samples, with different combinations of toxins found in isolates from the same individual (see Supplementary Table 1). The *cpa* gene was detected in all isolates, while *cpb2* was present in 10.7%; the *cpe* and *cpb* genes were undetected. Individuals carrying the *cpb2* gene showed no gastrointestinal symptoms. Notably, a strong association was found between the detection of the *cpb2* gene and diabetes and autoimmune diseases (adjusted OR 27.52: 95% IC 1.68–50.67).

## Discussion

The frequency of detection of *C. perfringens*, circulating toxins, and associated comorbidities in symptomatic and asymptomatic humans from a central region of Colombia was evaluated in this study, marking the first examination of its kind in the area. The presence of the bacterium in 19.3% of the sampled individuals and its significant association with diabetes and autoimmune diseases underscores the importance of conducting microgeographic studies and assessing host factors to anticipate and effectively address public health threats. Although the frequencies of detection in symptomatic individuals reported in this study (16.6%) (See Fig. 1A) are lower than those found in patients with diarrhea in Bogotá, Colombia (32.7%) [12], they fall within the range reported in other regions of the world. In Europe and Asia, the frequencies of *C. perfringens* infection range from 5 to 20% in antibiotic-associated diarrhea (AAD) cases and sporadic non-foodborne diarrhea [1, 7, 8]. *C. perfringens* is the second leading cause of foodborne bacterial illnesses [11, 18] in the United States.

While the percentage of *C. perfringens* detection in symptomatic patients in Colombia is high compared to studies in countries such as Germany (4.1%) [19] and India (8.6%) [20], it is similar to infection frequencies in China (13.8%) [21] and Iran (22.4%) [8]. These data significantly emphasize the relevant presence of *C. perfringens* in the central region of Colombia, highlighting the pressing need to consider the local context when interpreting detection frequencies in epidemiological studies. The variability in these data among different areas could be attributed to factors such as hygiene practices, environmental conditions, and demographic characteristics unique to each region, emphasizing the complexity of the epidemiology of *C. perfringens*.

Regarding the frequency of detection in asymptomatic individuals, our study revealed a rate of 21.2% **(**Fig. 1A**),** which is lower than that reported in healthy individuals in Germany (40.0%—20/50), North America (51.0%—22/43) [22], and northern Mexico (63.0%—126/200) [23]. Compared to studies in other countries, the low carriage frequency reported here could be attributed to differences in innate and acquired immune responses and to determinants of the host's genetic susceptibility to specific enteric infections [24].

It has been documented that prolonged exposure to *C. perfringens* or the sustained presence of small amounts of toxin in asymptomatic carriers has been reported to result in the detection of serum antitoxin antibodies [25–27]. In this context, it is plausible that antibodies generated against native toxins may inhibit *C. perfringens*-induced cytotoxicity [28]. This suggests the contribution of an enhanced immune defense mechanism in the population, offering a possible explanation for the observed local and global variations in detection frequencies and disease development.

Evaluation of the detection frequency in asymptomatic individuals provides a unique perspective highlighting the risk faced by this population, as they have a significantly higher propensity to experience episodes of invasive infection compared to uncolonized individuals [29]. Furthermore, the possibility of carrying toxigenic strains of *C. perfringens* reveals a potential risk, as they can serve as reservoirs and possible vehicles of transmission to other susceptible individuals [30].

Furthermore, future research should consider recent information from another study suggesting that the presence of *C. perfringens* at the intestinal level in asymptomatic individuals can trigger brain inflammation, oxidative stress, apoptosis, and cell damage [31].

This aspect becomes particularly relevant when exploring different age groups, especially in the presence of metabolic disorders.

Several studies have identified the presence of *C. perfringens* in diabetic patients with various complications, including wounds [32], bacteremia [33], gas gangrene [34], and necrotic enteritis [35–37]. Despite these findings, most investigations primarily focus on the molecular detection of the main toxins, neglecting to explore the association between accessory toxins and the presence of comorbidities as a risk factor for disease development [38].

In this study, conditional logistic regression, a crucial statistical tool in molecular epidemiological studies, was used to analyze the relationship between variables and an outcome of interest, such as the presence of a microorganism [39, 40]. This method controls for matching and comparison between dependent and independent variables, reducing bias and improving the precision of estimates of the effects of predictor variables in cross-sectional studies. Logistic regression facilitates the interpretation of results by minimizing the possibility that observed associations merely result from matching between variables rather than genuine causal relationships [40, 41].

The significant connection between the detection of the Cpb2 toxin-encoding gene and the presence of diabetes and autoimmune diseases (adjusted OR 27.52; 95% CI 1.68–50.67) suggests possible interactions between the microorganism and the host immune system.

The presence of impaired humoral immunity, defects in neutrophil function, and compromised T-cell response in hyperglycemic environments [42, 43], combined with tissue hypersensitivity to colonization factors due to reduced UDP-glucose [44] and alterations in intestinal motility [45], may contribute to the growth of toxigenic *C. perfringens* in diabetic patients. These findings underscore the importance of understanding the complex interactions between the microorganisms, toxin production, and the immune status of diabetic patients, with significant implications for preventing and treating potential associated complications.

Although we did not find other statistically significant associations, the most common underlying comorbidities in patients where *C. perfringens* was detected were cardiovascular diseases, diabetes, and hypercholesterolemia—findings similar to other studies [8, 21]. The dysfunction of the reticuloendothelial system in cardiovascular diseases and high cholesterol levels, precursors to the production of bile acids necessary for spore germination, may play a key role in colonization and infection by *C. perfringens* [46]. These data provide valuable information for guiding preventive measures in the colonized population.

Our study presents limitations that deserve acknowledgment for properly interpreting the findings. First, the limited sample size may not fully capture the diversity and prevalence of *C. perfringens*, particularly among symptomatic and asymptomatic populations. Furthermore, including a limited number of variables in our analysis left important aspects unexplored, which could influence the complete understanding of the epidemiology of this bacterium. Furthermore, our study's specific microgeographic representation may limit the results' generalizability to other regions or contexts.

The association of *C. perfringens* with food poisoning outbreaks and other tissue diseases highlights the importance of assessing the specific toxicity of toxins produced by this bacterium. Advancing the development of cell-based in vitro assays could provide a more accurate understanding of the actual impact of circulating toxigenic strains in both symptomatic and asymptomatic populations. This, in turn, would allow for a more informed and practical approach to controlling the prevalence of *C. perfringens*-associated disease [47].

In addition, genomic and phenotypic characterization of virulent strains carrying the *pfoA* gene and hypovirulent or “commensal” strains that encoded significantly fewer virulence factors and plasmids obtained from a more significant number of individuals and geographic areas would allow to deepen the epidemiological knowledge of *C. perfringens* in developing countries such as Colombia.

Finally, only patients with complete demographic profiles and risk factor data were included in the logistic regression. Consequently, the sample size was reduced for this analysis. We recommend that future studies make every effort to ensure complete registration to avoid excluding patients from the analysis.

In conclusion, this study explores the results within a regional context, delving into factors associated with patients experiencing intestinal symptoms. It underscores the significance of detection in individuals without intestinal symptoms. Moreover, it indicates future directions, emphasizing the urgent need to explore microbial dynamics within the human intestine further and consider specific preventive measures for the at-risk population [48]. Therefore, people must cook food above 60 °C and refrigerate it to −5 °C within 2 h after cooking. In addition, refrigerated foods should be reheated to temperatures above 60ºC before consumption, allowing the vegetative cell's destruction and controlling the source and cross-contamination [49].

Despite its crucial public health importance, there are limited studies on the epidemiology of *C. perfringens* in Colombia [12], and its association with food poisoning outbreaks remains unknown due to the absence of routine tests to identify this microorganism. The findings presented provide a first overview of the frequency of detection of *C. perfringens*; however, larger-scale epidemiological studies in Colombia are needed to provide a more complete picture. These expanded studies would offer valuable insights into public health management and implementing preventive strategies.

### Supplementary Information

Additional file 1: Figure Supplementary 1. Sampling areas in the Department of Boyacá Colombia. The hospitals included in the study are part of the municipalities highlighted in orange.

Additional file 2.

Additional file 3.

## Data Availability

No datasets were generated or analysed during the current study.
