# Supplementary figures and images for: Clostridium perfringens in central Colombia: frequency, toxin genes, and risk factors
Source: Gut Pathog. 2024 Jul 4;16:32. doi: 10.1186/s13099-024-00629-5 (PMC11225238; doi:10.1186/s13099-024-00629-5)

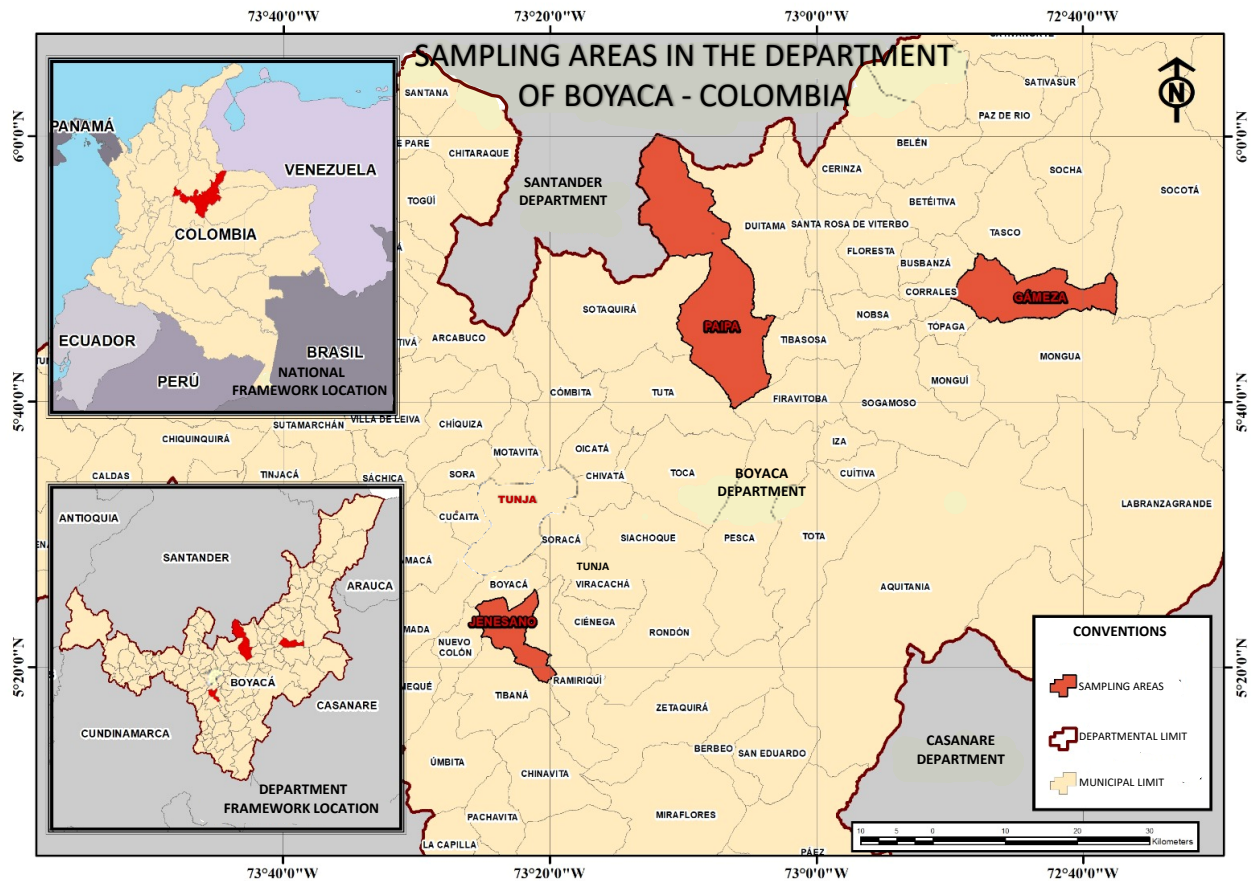

Supplement: Supplementary file 1 — Additional file 1: Figure Supplementary 1. Sampling areas in the Department of Boyacá Colombia. The hospitals included in the study are part of the municipalities highlighted in orange. [file 13099_2024_629_MOESM1_ESM.pdf]
