# Supplementary material for: Clostridium perfringens in central Colombia: frequency, toxin genes, and risk factors
Source: Gut Pathog. 2024 Jul 4;16:32. doi: 10.1186/s13099-024-00629-5 (PMC11225238; doi:10.1186/s13099-024-00629-5)
Supplement: Supplementary file 3 — Additional file 3. [file 13099_2024_629_MOESM3_ESM.docx]

**Supplementary table 2.** Demographic profile and risk factors associated with *C. perfringens* detection

|  | ***C. perfringens* detection (*n=*77)** | | | | **Univariate analysis** | | ***p-*value** | **Multivariate analysis** | |  |  |
| --- | --- | --- | --- | --- | --- | --- | --- | --- | --- | --- | --- |
| **Variables** | **Negative**  ***n=*62** | | **Positive**  ***n=*15** | | **OR** | **95%CI** |  | **OR**^a^ | **95%CI** | ***p-*value** |  |
| **Age, median (IQR)** | 57.5  (11.5) | | 60.0  (11.1) | | 1.03^*^ | 0.98-1.08 | 0.188 | 0.99^*^ | 0.92-1.07 | 0.884 |  |
|  | ***n*** | **(%)** | ***n*** | **(%)** |  |  |  |  |  |  |  |
| **Animals at home** |  |  |  |  |  |  |  |  |  |  |  |
| No | 23 | 29.9 | 9 | 11.7 | Reference | |  | Reference | |  |  |
| Yes | 39 | 50.6 | 6 | 7.8 | 0.37 | 0.11-1.19 | 0.098 | 0.35 | 0.08-1.56 | 0.172 |  |
| **Antibiotic use** |  |  |  |  |  |  |  |  |  |  |  |
| No | 32 | 41.6 | 11 | 14.3 | Reference | |  | Reference | |  |  |
| Yes | 30 | 39.0 | 4 | 5.2 | 0.38 | 0.11-1.35 | 0.137 | 0.37 | 0.08-1.55 | 0.175 |  |
| **Access to drinking water** |  |  |  |  |  |  |  |  |  |  |  |
| No | 11 | 14.3 | 3 | 3.9 | Reference | |  | Reference | |  |  |
| Yes | 51 | 66.2 | 12 | 15.6 | 0.78 | 0.18-3.29 | 0.740 | 1.07 | 0.17-6.71 | 0.939 |  |
| **Hypertension** |  |  |  |  |  |  |  |  |  |  |  |
| No | 43 | 55.8 | 7 | 9.1 | Reference | |  | Reference | |  |  |
| Yes | 19 | 24.7 | 8 | 10.4 | 2.13 | 0.67-6.77 | 0.196 | 0.87 | 0.20-3.71 | 0.852 |  |
| **Gastritis** |  |  |  |  |  |  |  |  |  |  |  |
| No | 56 | 72.7 | 13 | 16.9 | Reference | |  | Reference | |  |  |
| Yes | 6 | 7.8 | 2 | 2.6 | 1.43 | 0.25-7.94 | 0.678 | 2.21 | 0.32-15.22 | 0.417 |  |
| **Diabetes/Autoimmune disease** |  |  |  |  |  |  |  |  |  |  |  |
| No | 56 | 72.7 | 12 | 15.6 | Reference | |  | Reference | |  |  |
| Yes | 6 | 7.8 | 3 | 3.9 | **4.66** | **1.19-18.26** | **0.027** | **8.41** | **1.32-35.59** | **0.024** |  |
| **Hypothyroidism** |  |  |  |  |  |  |  |  |  |  |  |
| No | 57 | 74.0 | 13 | 16.9 | Reference | |  | Reference | |  |  |
| Yes | 5 | 6.5 | 2 | 2.6 | 1.75 | 0.30-10.06 | 0.528 | 1.53 | 0.20-11.38 | 0.672 |  |
| **Hypercholesterolemia** |  |  |  |  |  |  |  |  |  |  |  |
| No | 53 | 68.8 | 12 | 15.6 | Reference | |  | Reference | |  |  |
| Yes | 9 | 11.7 | 3 | 3.9 | 1.45 | 0.35-5.89 | 0.598 | 2.61 | 0.46-14.81 | 0.278 |  |
| **Symptoms** |  |  |  |  |  |  |  |  |  |  |  |
| No | 37 | 48.1 | 10 | 13.0 | Reference | |  | Reference | |  |  |
| Yes | 25 | 32.5 | 5 | 6.5 | 0.74 | 0.22-2.42 | 0.619 | 1.31 | 0.27-6.22 | 0.682 |  |

Abbreviations: IQR: interquartile range

^*^ For variable age the increase of years is the reference category
